# Supplementary material for: Melting of recycled ancient crust responsible for the Gutenberg discontinuity
Source: Nat Commun. 2020 Jan 10;11:172. doi: 10.1038/s41467-019-13958-w (PMC6954225; doi:10.1038/s41467-019-13958-w)
Supplement: Supplementary file 1 — Supplementary Information [file 41467_2019_13958_MOESM1_ESM.pdf]

Supplementary Information

Melting of recycled ancient crust responsible for the Gutenberg discontinuity

Jia Liu et al.

List for Supplementary Figures

Supplementary Figure 1. The comparison of the basic geochemical characteristics for the whole petit-spot basalts and the selected ones in this study.
Supplementary Figure 2. $\delta^{26}Mg$ vs. $\delta^{25}Mg$ for the petit-spot basalts in the NW pacific and the USGS standards.
Supplementary Figure 3. The comparison of $\delta^{26}Mg$, the loss on ignition (LOI) and trace elemental ratios for basalts from Site A.

List for Supplementary Data

Supplementary Data 1. The major element composition of the petit-spot basalts in the NW Pacific.
Supplementary Data 2. Trace element concentrations of the petit-spot basalts in the NW Pacific in ppm.
Supplementary Data 3. The Sr-Nd-Pb isotopic composition of the petit-spot basalts in the NW Pacific.
Supplementary Data 4. The Mg isotopic compositions relative to DSM-3 of petit-spot basalts in NW Pacific.

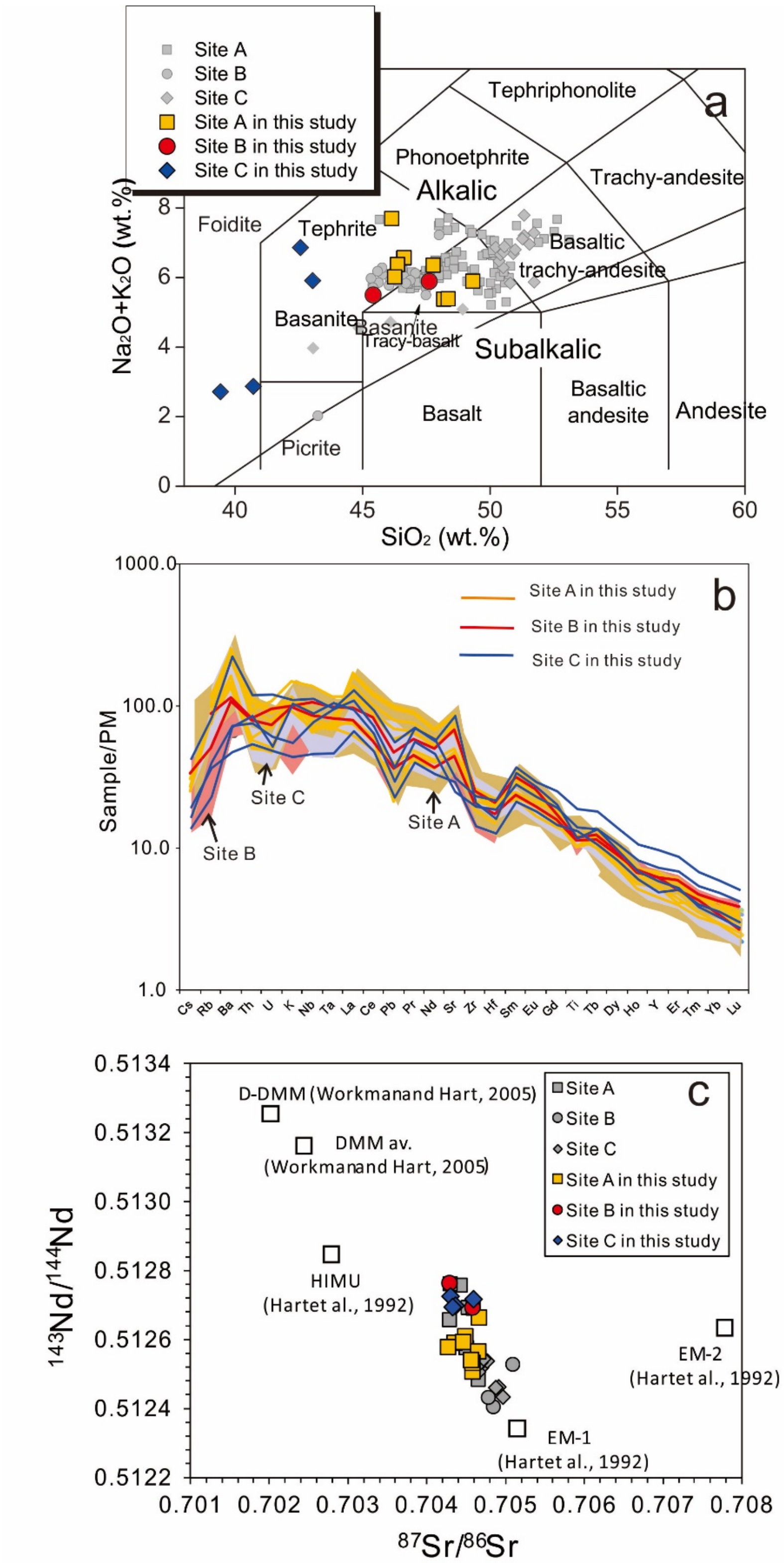

Supplementary Figure 1. The comparison of the basic geochemical characteristics for the whole petit-spot basalts and the selected ones in this study. a, the total alkali vs. SiO_2 for the petits-pot basalts in the NW pacific. The gray data are from ref.[1] and the references therein. The colored samples were selected for Mg isotopes and other geochemical analyses. The framework is after ref.[2]. **b**, the primitive-mantle normalized trace element patterns. The primitive mantle value is from ref. [3]. **c**, the Sr-Nd isotopic covariation diagram. The data for the selected samples in this study

are from ref. [1,4] and this study (see Supplementary Data 1,2,3).

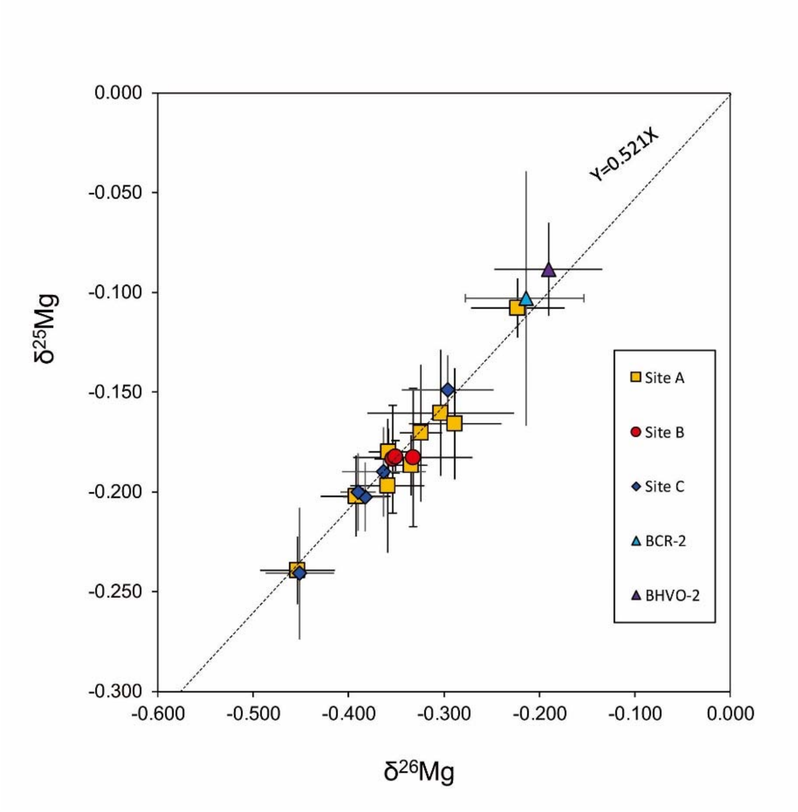

Supplementary Figure 2. δ^{26}Mg vs. δ^{25}Mg for the petit-spot basalts in the NW pacific and the USGS standards. It is noted that all data distribute along the terrestrial equilibrium mass fractionation line with a slope of 0.521 (ref. [5]).

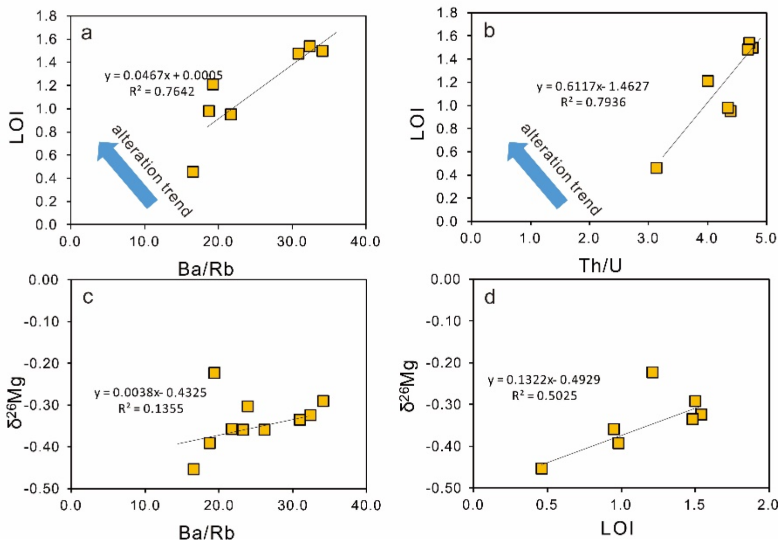

Supplementary Figure 3. The comparison of δ^{26}Mg, the loss on ignition (LOI) and trace elemental ratios for basalts from Site A. a, LOI vs. Ba/Rb, the blue arrow points to the trend expected for seawater alteration. **b,** LOI vs. Th/U, the blue arrow points to the trend expected for

seawater alteration. **c**. the comparison of δ^{26}Mg with Ba/Rb ratio. **d**, the comparison of δ^{26}Mg with LOI. The δ^{26}Mg values of the normal mantle (average of global MORB and OIBs) is from ref. [6].

Supplementary References

1. Machida, S. *et al.* Petit-spot geology reveals melts in upper-most asthenosphere dragged by lithosphere. *Earth Planet. Sci. Lett.* **426,** 267–279 (2015).
2. Le Maitre, R.W., 2002. Igneous Rocks A Classification and Glossary of Terms, second ed. Cambridge.
3. McDonough, W. F. & Sun, S.-s. The composition of the Earth. *Chem. Geol.* **120,** 223–253 (1995).
4. Machida, S., Hirano, N. & Kimura, J. Evidence for recycled plate material in Pacific upper mantle unrelated to plumes. *Earth Planet. Sci. Lett.* **73,** 3028–3037 (2009).
5. Young E. D. and Galy A. The Isotope Geochemistry and Cosmochemistry of Magnesium. Rev. Mineral. Geochem. 55, 197-230 (2004).[1]
6. Teng F-Z, et al. (2010) Magnesium isotopic composition of the Earth and chondrites. Geochim Cosmochim Acta 74(14):4150–4166.
