# Supplementary material for: Melting of recycled ancient crust responsible for the Gutenberg discontinuity
Source: Nat Commun. 2020 Jan 10;11:172. doi: 10.1038/s41467-019-13958-w (PMC6954225; doi:10.1038/s41467-019-13958-w)
Supplement: Supplementary file 3 — Description of Additional Supplementary Files [file 41467_2019_13958_MOESM3_ESM.pdf]

Description of Additional Supplementary Files

File Name: Supplementary Data 1
Description: The major element composition of the petit-spot basalts in the NW Pacific.

File Name: Supplementary Data 2
Description: Trace element concentrations of the petit-spot basalts in the NW Pacific in ppm.

File Name: Supplementary Data 3
Description: The Sr-Nd-Pb isotopic composition of the petit-spot basalts in the NW Pacific.

File Name: Supplementary Data 4
Description: The Mg isotopic compositions relative to DSM-3 of petit-spot basalts in NW Pacific.
